# Should we stop referring to the pandemic of antimicrobial resistance as silent?

**DOI:** 10.1093/jacamr/dlae018

**Published:** 2024-02-07

**Authors:** Miroslav Sirota

**Affiliations:** Department of Psychology, University of Essex, Wivenhoe Park, Colchester CO4 3SQ, UK

## Abstract

**Background:**

Referring to the ongoing antimicrobial resistance crisis as a ‘silent’ pandemic has gained popularity, but there are mixed views on whether such a phrase should be used in public health communication. Some researchers have argued that using the term ‘silent pandemic’ may lower the perceived threat and hinder mobilization efforts to tackle the problem.

**Objectives:**

I investigated the impact of the phrase ‘silent pandemic’ on perceived threat levels and mobilization intentions.

**Methods:**

In three experiments (*n* = 1677), participants from the UK’s general adult population were randomly allocated to either a ‘pandemic’ or ‘silent pandemic’ condition, where the different terms were embedded in statements (Experiment 1) or brief information materials (Experiments 2 and 3). The term ‘silent pandemic’ was also presented with a brief description of its intended meaning (Experiment 3). The participants expressed their perception of the threat and their mobilization intentions.

**Results:**

In Experiments 1 and 2, referring to the pandemic as silent did not significantly affect the perceived threat (Cohen’s *d* = −0.06; Cohen’s *d* = 0.08, respectively) or mobilization intentions (Cohen’s *d* = −0.07; Cohen’s *d* = 0.11, respectively). However, in Experiment 3, the term ‘silent pandemic’ decreased the perceived threat and mobilization intentions (Cohen’s *d* = 0.27; Cohen’s *d* = 0.35, respectively).

**Conclusions:**

Describing the pandemic as ‘silent’ yielded no measurable effects on perceived threat and mobilization intentions but it showed depreciating effects when accompanied by its intended meaning. Taken together, it is advisable to avoid the term.

## Introduction

Antimicrobial resistance (AMR) is an alarming global health threat.^1^ AMR contributes to the deaths of millions of people every year, inflicts substantial economic damage and exacerbates health inequalities worldwide.^2–4^ Recognizing the urgency of the situation, the WHO identified public awareness and education as a top research priority in the recent global research agenda for AMR in human health.^5^ However, effectively communicating this threat to the public remains a critical challenge in raising public awareness.

The term ‘silent pandemic’ was recently used in an attempt to raise awareness about the ongoing pandemic in public and academic communication. Indeed, a Google search returned 56 700 results of the use of the expression ‘silent pandemic’ in association with AMR at the time of writing this article (based on a Google search conducted on 3 July 2023 using the query: ‘silent pandemic’ AND (‘antimicrobial* resistance’ OR ‘AMR’ OR ‘antibiotic* resistance’)). The WHO^6^ and the ECDC^7^ routinely use this term in their communication with the public. A recent documentary, which aimed to convey the extent of the AMR crisis to the public, was named simply ‘Silent Pandemic’.^8^ This term is also popular in academic discourse.^9,10^ In the Web of Science database, using the same query as above, the expression ‘silent pandemic’ in association with AMR can be traced to an article published in 2018^11^ and became popular during the COVID-19 pandemic.

However, it is unclear whether this term is suitable and effective for public health communication. Some authors have opposed the use of this expression.^12^ They have reasoned that the modifier ‘silent’ might imply that the ongoing threat is not worthy of attention and may interfere with the intention to act and mobilize support for the actions needed to address the pandemic. Such interpretation and consequences are not aligned with its intended meaning. The silent pandemic should, in fact, imply a lack of public awareness and understanding of resistance while representing a concealed threat to public health, which spreads unnoticed. All of these interpretations invite an interpreter to recognize AMR as a pressing issue, with a high level of threat requiring urgent action. Both types of interpretations have the potential to alter risk perception and, thus, downstream effects related to antibiotic-relevant decision-making.^13,14^

Since the communication effect is not clear, I tested whether the phrase ‘silent pandemic’, in contrast to simply ‘pandemic’, affects the perceived threat of AMR and the associated mobilization intentions. Three hypotheses were postulated. First, advocates of the term ‘silent’ pandemic anticipate that it would enhance the perceived threat of the ongoing crisis and encourage public involvement in safeguarding antimicrobials. Second, opponents argue that using this term would downplay the seriousness of the pandemic and hinder mobilization efforts. Finally, it is plausible that the choice of words has no discernible effect on perceptions and mobilization intentions.

## Experiment 1

### Methods

#### Ethics

This research was conducted in accordance with the Declaration of Helsinki and institutional guidelines. Approval was obtained from the departmental ethics committee at the University of Essex (Reference Numbers: ETH1920-0862 for Experiments 1 and 2; ETH2324-0194 for Experiment 3). Written informed consent was obtained from all study participants. Their privacy and confidentiality were protected throughout the research process. Data collection followed ethical guidelines, ensuring the anonymity and confidentiality of participants’ information. Relevant data protection regulations were strictly followed.

#### Participants

I recruited 426 participants from the general adult population of the UK using the online panel Prolific in June 2023. Based on *a priori* power analysis, 352 participants were required to be able to detect a small effect (Cohen’s *d* = 0.3) assuming α = 0.05 and 1−β = 0.80, for an independent-samples *t*-test. However, the sample size exceeded the target to account for potential attrition. Only participants who were 18 years of age or older, residing in the UK, having English as their first language, and holding UK citizenship were eligible to participate. Following the *a priori* exclusion criterion, three participants were excluded because they failed to pass the instructional manipulation check. The recruited sample was sex balanced.

The analytical sample consisted of 423 participants (ages ranging from 19 to 79 years, mean = 44.1, SD = 14.3 years). Among the participants, 48.0% identified as female, 51.5% as male, and 0.5% selected another option for gender. The distribution of the participants’ education levels was as follows: 0.9% did not complete high school, 35.9% completed high school, 44.7% completed a college degree, 15.6% completed a Master’s degree, and 2.8% completed a PhD or other professional degree. The most common occupational categories were: management, professional and related occupations (29.1%); retired (11.6%); unemployed (9.7%); government occupations (9.0%); service occupations (8.5%); and sales and office occupations (7.8%). The remaining participants were in other, less common occupations. Regarding ethnic groups, the participants were predominantly white (88.4%), with smaller proportions belonging to black (2.8%), Asian (3.8%), mixed (4.5%) and other (0.5%) ethnic groups.

#### Design, materials and procedure

Upon providing informed consent, participants were asked to assess statements regarding AMR. In a between-subjects experiment, participants were randomly assigned to either the pandemic condition (*n* = 211), where AMR was referred to as an ongoing ‘pandemic’ or the silent pandemic condition (*n* = 212), where AMR was described as an ongoing but ‘silent’ pandemic. The key manipulated terms were in bold type to direct the attention of participants to the terms [see Supplementary Materials (available as Supplementary data at *JAC-AMR* Online)]. The study employed a single-blind randomized controlled trial design, with randomized participant allocation using the Qualtrics built-in randomizer, which automatically utilized the Mersenne Twister algorithm.^15^ With these statements, participants evaluated the level of threat (e.g. ‘The silent pandemic of antimicrobial resistance is an exceptionally serious health threat’) and their intention to raise awareness (e.g. ‘I am willing to raise more awareness about the silent pandemic of antimicrobial resistance’) using a 7-point Likert scale ranging from 1 = *Strongly Disagree* to 7 = *Strongly Agree*. The presentation order of the statements was randomized. They were then asked to complete the sociodemographic information and were debriefed after the study (see Supplementary Materials).

##### Statistical analyses

An independent samples *t*-test was used to test the effect of the term on the dependent variables using the R programming language.^16^ Since not rejecting the null hypothesis does not logically entail accepting the null hypothesis, I quantified the evidence to support the null or alternative hypothesis by computing a Bayes factor (BF) with default medium prior scales of 0.707 using the ‘BayesFactor’ package in R. In generic terms, a BF is the ratio of the probability of the data given model A (e.g. H_1_) to the probability of the data given model B (e.g. H_0_). Thus, the BF expresses the ratio of the marginal likelihood of the data under model A (e.g. H_1 effect model_) and model B (e.g. H_0 intercept-only model_) and allows us to quantify how many more times the data are likely to occur under H_1_ compared with H_0_ or vice versa. For example, a BF_01_ value of 5 indicates that the data are five times more likely to occur under H_0_. The BF values may also be interpreted as evidence categories; for example, values of BF_01_ between 3 and 10 indicate *substantial evidence* to support the null hypothesis.^17^ Additionally, I conducted a series of exploratory, not pre-registered, interaction analyses with the variables of age, gender (male and female) and education (dichotomized as ‘high-school or lower’ education versus ‘college and higher’ education) as possible moderators entered with the manipulation in separate linear regressions.

### Results and discussion

**Figure 1.**
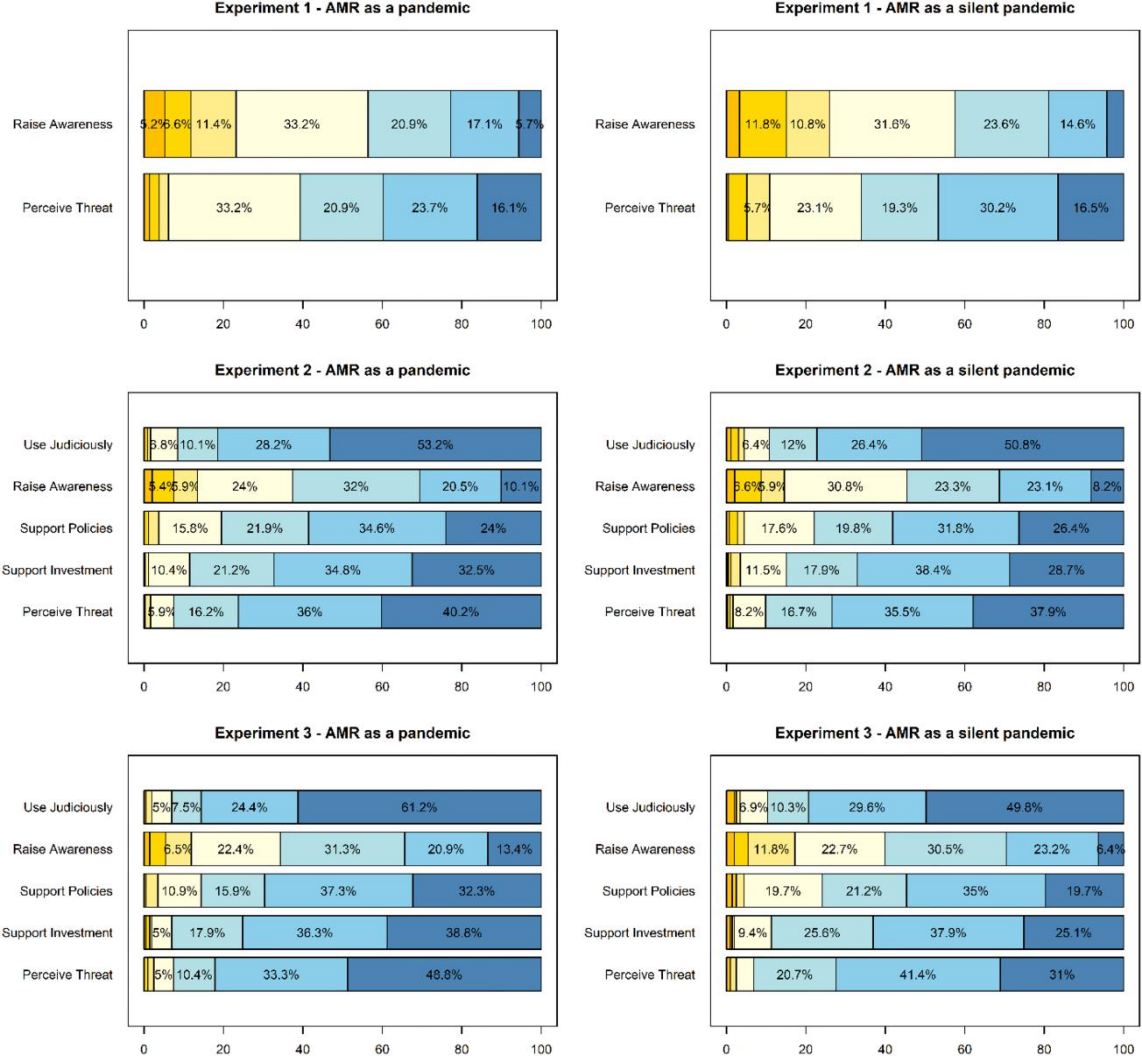
The effect of AMR presented as a silent pandemic (versus pandemic) on its perceived threat and mobilization intentions. The Likert scale values on the graph range from ‘Strongly disagree’ on the left, represented in a dark yellow, to ‘Strongly agree’ on the right, depicted in a dark blue. The numerical values below 5% are not shown for reasons of presentation.

**Figure 2.**
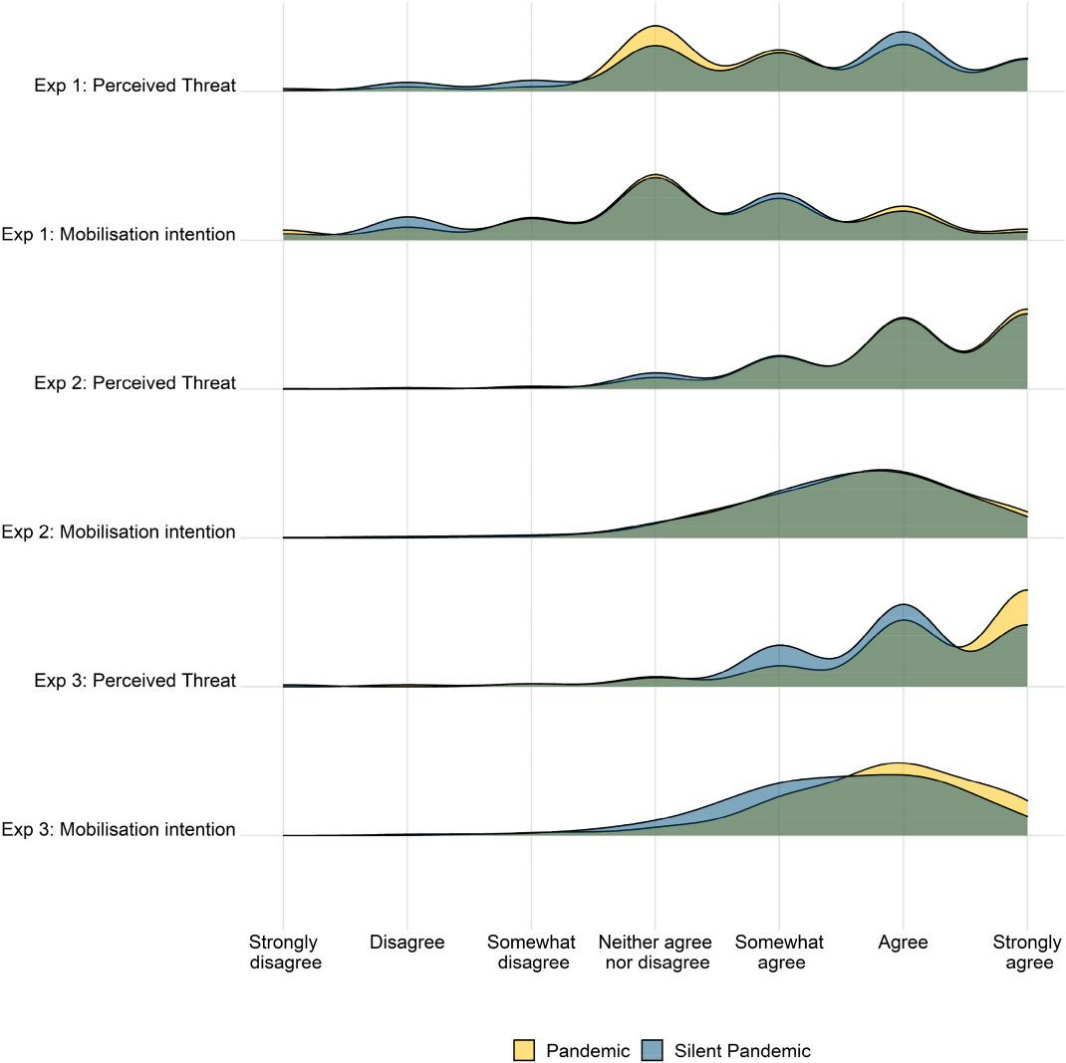
The effect of information about AMR presented as a silent pandemic (versus pandemic) on its perceived threat and mobilization intentions.

Participants in the silent pandemic condition perceived a similarly high level of threat associated with AMR (mean = 5.1, SD = 1.4) compared with those in the pandemic condition (mean = 5.1, SD = 1.3; see also Figures 1 and 2). The difference between the conditions was not statistically significant and was very small: *t*(421) = −0.57, *P* = 0.568, Cohen’s *d* = −0.06. The BF analysis yielded substantial relative evidence to support the null hypothesis, BF_01_ = 7.9.

Similarly, participants in the silent pandemic condition expressed similarly strong intentions to raise awareness about the issue (mean = 4.2, SD = 1.4) compared with those in the pandemic condition (mean = 4.3, SD = 1.5; see Figure 1). There were no significant and only minimal descriptive differences, between the two conditions: *t*(421) = −0.75, *P* = 0.456, Cohen’s *d* = −0.07 (see Figure 2). The BF analysis yielded substantial relative evidence to support the null hypothesis, BF_01_ = 7.1.

This means that the term ‘silent pandemic’ (versus ‘pandemic’) had no detectable effects on the public’s perceived threat of AMR nor on their intentions to raise awareness about the pandemic.

Finally, in exploratory moderation analyses, none of the moderators—age, gender or education levels—interacted with the perceived threat or mobilization intentions (see Table 1).

**Table 1.** The interaction effect of silent pandemic (versus pandemic) with age, gender and education on perceived threat and mobilization intentions (exploratory moderation analyses)

Experiment	Moderator	Dependent variable	Estimate (*b*)	*P* value
Experiment 1	Age	Threat	−0.005	0.564
		Mobilization	0.000	0.980
	Gender	Threat	0.115	0.663
		Mobilization	−0.155	0.586
	Education	Threat	−0.074	0.786
		Mobilization	−0.034	0.907
Experiment 2	Age	Threat	0.016	0.002
		Mobilization	0.007	0.144
	Gender	Threat	0.253	0.070
		Mobilization	0.043	0.728
	Education	Threat	0.011	0.940
		Mobilization	−0.026	0.837
Experiment 3	Age	Threat	−0.010	0.189
		Mobilization	−0.003	0.592
	Gender	Threat	0.069	0.737
		Mobilization	0.003	0.987
	Education	Threat	0.229	0.277
		Mobilization	0.230	0.199

The threshold α value was adjusted to 0.003 to account for the number of comparisons (18), corresponding to 0.05/18. Estimates (b) denote regression coefficients for the interaction terms in multiple linear regression models.

This study has some limitations. First, the manipulation employed in this study was decontextualized as it focused on the assessment of single-sentence statements. The term ‘silent pandemic’ is typically used in the context of public health messaging providing more information about the ongoing pandemic. Second, a positive/negative effect might exist, but it is smaller than what this experiment was powered to detect (Cohen’s *d* of approximately 0.3). Third, I measured a specific intention to act, namely to raise awareness. It must be recognized that there are numerous other actions the public can undertake, which may be hindered by suboptimal communication about the crisis.

## Experiment 2

Experiment 2 was conducted to address these limitations. In this experiment, I integrated the manipulation into the information provided about AMR adapted from the WHO website. I also doubled the sample size and expanded the range of intentions to act. Specifically, I measured five intentions to act, encompassing expressions of support for systemic changes such as regulatory policies, in addition to individual actions such as the judicious use of antibiotics.

### Methods

#### Participants

I recruited a new sample of 853 participants from the general adult population of the UK using the online panel Prolific in June 2023. Based on *a priori* power analysis, 788 participants were required to be able to detect a small effect (Cohen’s *d* = 0.2) assuming α = 0.05, and 1−β = 0.80, for an independent-samples *t*-test. This number was adjusted upward to account for potential attrition. The same eligibility and exclusion criteria were applied as in Experiment 1. Following the *a priori* exclusion criterion, three participants were excluded because they failed to pass the instructional manipulation check. The recruited sample was sex-balanced.

The analytical sample consisted of 850 participants (ages ranging from 19 to 78 years, mean = 42.7, SD = 13.6 years). Among the participants, 50.7% identified as male, 48.6% as female, and 0.7% selected another gender option. The distribution of the participants’ education levels varied: 1.4% did not complete high school, 38.4% completed high school, 42.1% completed a college degree, 14.8% completed a Master’s degree, and 3.3% completed a PhD or other professional degree. The most common occupational categories were: management, professional and related occupations (29.6%); sales and office occupations (12.9%); retired (9.2%); unemployed (8.1%); government occupations (6.7%); and service occupations (6.2%). The remaining participants were in other, less common occupations. Regarding ethnic groups, the participants were predominantly white (90.0%), with smaller proportions belonging to black (2.5%), Asian (5.4%), mixed (1.8%) and other (0.4%) ethnic groups.

#### Design, materials and procedure

Upon providing informed consent, the participants were randomly assigned to either the pandemic condition (*n* = 425), where AMR was referred to as an ongoing ‘pandemic’ or the silent pandemic condition (*n* = 425), where AMR was described as an ongoing ‘silent’ pandemic. The key manipulated terms were in bold type (see Supplementary Materials). In both conditions, participants read a short informational text about AMR, its causes and consequences. This was adapted from the WHO webpage (see Supplementary Materials).

After reading the text, the participants evaluated the level of threat associated with AMR (e.g. ‘The silent pandemic of antimicrobial resistance is an exceptionally serious health threat.’) and their mobilization intentions using a 7-point Likert scale ranging from 1 = *Strongly Disagree* to 7 = *Strongly Agree*. The mobilization intentions included support for systemic-level actions such as financial investment and support for stricter policies (i.e. ‘I believe that the government should invest more money in addressing the issue of antimicrobial resistance,’ and ‘I support strict policies that promote the judicious use of antimicrobials’). They also included individual-level actions such as raising awareness and judicious use of antimicrobials (i.e. ‘I am willing to actively raise more awareness about antimicrobial resistance,’ and ‘I intend to use antimicrobials only as prescribed by a healthcare professional’). The participants were then asked to complete the sociodemographic information and were debriefed after the study.

##### Statistical analyses

The same analytical approach was adopted as in Experiment 1.

### Results and discussion

The term ‘silent pandemic’ (versus ‘pandemic’) had no noticeable descriptive effect on the public’s perceived level of the threat of AMR (see Figures 1 and 2). Indeed, participants in the pandemic condition perceived a similarly high level of threat (mean = 6.1, SD = 1.0) to those in the silent pandemic condition: mean = 6.0, SD = 1.1, *t*(848) = 1.21, *P* = 0.226, Cohen’s *d* = 0.08. The BF analysis yielded substantial evidence to support the null hypothesis compared with the alternative hypothesis, BF_01_ = 6.3.

Similarly, the term ‘silent pandemic’ (versus ‘pandemic’) did not lead to any visible differences in agreement with systemic-level changes such as support for larger investments in resolving AMR, support for stricter policies regulating antimicrobial use, intentions to raise awareness about the issue and the judicious use of antimicrobials (see Figure 2). As pre-registered, I first checked whether this set of variables could be treated as a unitary construct. In this case, the scree plot yielded a one-factor solution, with all items loaded highly on one factor (0.54–0.72) while the one-factor model generated an acceptable fit [Tucker–Lewis Index (TLI) = 0.88, root mean square error of approximation (RMSEA) = 0.13, 90% CI (0.09–0.17)]. The internal consistency of the average index was acceptable (Cronbach’s α = 0.73). Participants in the pandemic condition expressed similar mobilization intentions (mean = 5.6, SD = 0.9) to those in the silent pandemic condition (mean = 5.5, SD = 1.0). Again, the difference between the conditions was not statistically significant: *t*(848) = 1.64, *P* = 0.101, Cohen’s *d* = 0.11. The BF analysis yielded substantial relative evidence to support the null hypothesis, BF_01_ = 3.5.

Finally, in exploratory moderation analyses, only age interacted with the perceived threat—with younger people perceiving a lower level and older people a higher level of threat for ‘silent pandemic’ relative to ‘pandemic’. None of the other interactions were significant (see Table 1). Since this was the only moderation effect detected in the context of exploratory analyses and not replicated in the other two experiments, it is important to avoid overinterpreting this finding. Future research should consider age as a potential moderator.

In both experiments, the manipulations used the simple term ‘silent’ without additional explanations. This had several reasons. First, the term is usually used without accompanying explanations, relying on its self-explanatory nature. Second, using only the term allows participants to generate their associations and interpretations of the term. However, one could also argue that such manipulation is weak and there might be communication instances, in which the intended meaning of the term is explained. Therefore, I conducted a follow-up experiment.

## Experiment 3

Experiment 3 was conducted to test a stronger manipulation, in which participants read the intended meaning of the term ‘silent pandemic’. The explanation was embedded into the information provided about AMR adapted from the website of the WHO.

### Methods

#### Participants

I recruited a new sample of 404 participants from the general adult population of the UK using the online panel Prolific in October 2023. The power calculation, stopping rule and exclusion criteria were the same as in Experiment 1. The eligibility criteria were the same as well, with one exception—participants could not take part in the previous two experiments reported here. Following the *a priori* exclusion criterion (i.e. failed instructional reading check), none of the 404 participants who completed the study was excluded. The recruited sample was sex-balanced.

The analytical sample consisted of 404 participants (ages ranging from 19 to 82 years, mean = 42.4, SD = 13.5 years). Among the participants, 49.0% identified as female, 50.7% as male, and 0.2% selected another option for gender. The distribution of the participants’ education levels was as follows: 0.7% did not complete high school, 38.1% completed high school, 45.0% completed a college degree, 13.6% completed a Master’s degree, and 2.5% completed a PhD or other professional degree. The most common occupational categories were: management, professional and related occupations (36.4%); sales and office occupations (12.6%); service occupations (8.4%); retired (8.9%); unemployed (6.9%); and government occupations (5.4%). The remaining participants were in other, less common occupations. Regarding ethnic groups, the participants were predominantly white (88.4%), with smaller proportions belonging to black (2.8%), Asian (3.8%), mixed (4.5%) and other (0.5%) ethnic groups.

#### Design, materials and procedure

The design, materials, dependent measures and procedure were identical to those used in Experiment 2. There were two changes. First, the term silent pandemic was explained in the silent pandemic condition: ‘Antimicrobial resistance is termed a “silent pandemic” because it spreads quietly and gradually around the world, receiving less attention than it should compared with other pandemics, despite being equally harmful to people’s health.’ Second, a reading recall question was also asked on a separate screen following the answer to the dependent measures to probe the text memory of the text (see Supplementary Materials). This was a multiple-choice question (‘Which of the following statements was mentioned in the text you just read?’) with four options (e.g. ‘Antimicrobial resistance is termed a “silent pandemic” because it quietly and gradually spreads around the world receiving less attention than it should.’). I used the same dependent measures. The mobilization intentions created an average index for which internal consistency was acceptable (Cronbach’s α = 0.73). The participants were then asked to complete the sociodemographic information and were debriefed after the study.

##### Statistical analyses

The same analytical approach was adopted as in Experiment 2.

### Results and discussion

Participants in the pandemic condition perceived a significantly higher level of threat (*n* = 201, mean = 6.2, SD = 1.0) than those in the silent pandemic condition: *n* = 203, mean = 5.9, SD = 1.0, *t*(402) = 2.71, *P* = 0.007, Cohen’s *d* = 0.27. The BF analysis yielded substantial evidence to support the alternative compared to the null hypothesis, BF_10_ = 3.7. The effect was even more pronounced for those who recalled the correct text in a subsequent recall question: *t*(288) = 3.49, *P* < 0.001, Cohen’s *d* = 0.44. Thus, the explained term ‘silent pandemic’ (versus ‘pandemic’) yielded a lower perceived level of the threat of AMR (see Figures 1 and 2).

Participants in the pandemic condition also expressed higher scores on mobilization intentions (mean = 5.8, SD = 0.9) compared with participants in the silent pandemic condition (mean = 5.5, SD = 0.9; see Figures 1 and 2). This difference was statistically significant: *t*(402) = 3.48, *P* < 0.001, Cohen’s *d* = 0.35. The BF analysis yielded substantial relative evidence to support the alternative hypothesis, BF_10_ = 35.3. The effect was only slightly more pronounced for those who recalled the correct text in a subsequent recall question: *t*(288) = 3.07, *P* = 0.002, Cohen’s *d* = 0.39.

Finally, none of the tested sociodemographic moderators yielded significant interactions with the manipulation (see Table 1).

## General discussion

Two well-powered experiments found that using the term ‘silent pandemic’ as opposed to ‘pandemic’ does not elicit a significant change in the perceived threat of AMR. Also, it does not hinder the public’s mobilization to support systemic changes or engage in individual behaviours, such as raising awareness or practising judicious use of antimicrobials. Consequently, and contrary to the expectations of the term’s adopters^7^ as well as its opponents,^12^ the utilization of this term had no substantial consequences on the public’s perception. However, as shown in Experiment 3, once the expression’s intended meaning was presented to participants they rated the perceived threat and their mobilization intentions lower compared with the ‘pandemic’ term group.

The implications of this research are 2-fold. First, public health communicators are presented with a choice regarding the usage of the term ‘silent’ pandemic when referring to AMR. The findings suggest that the impact of this particular phrase may be consequential only when coupled with its intended meaning. In terms of a cost–benefit analysis, however, the absence of intended benefits associated with the term ‘silent pandemic’, coupled with the presence of documented depreciating impacts, suggests that the costs of using this term outweigh any potential benefits. Consequently, it is recommended to refrain from using this term. Second, these findings underscore the need for a more evidence-based approach to public health communication in general. As the challenges posed by AMR continue to unfold, it becomes increasingly crucial to carefully consider how we communicate information about this crisis to the public.^18^ Behavioural science offers many tools that can form the basis of evidence-based public health communication.^19^ By adopting an evidence-based approach, informed by research such as this, we can make conscious decisions regarding the selection of words, messages and strategies that effectively convey the gravity of the issue while promoting public understanding and engagement.

Several limitations of the reported experiments should be considered. The participants in these experiments were recruited from the general adult population of the UK. Participants from other countries, specific subgroups and cultural contexts may be more sensitive to word choices that modify pandemics. Future research should investigate this further. Thus, our conclusion has limited generalizability to populations of other countries. Second, behavioural intentions, as measured here, are good but far from perfect predictors of actual behaviours.^20^ Future research could examine the consequences of such wordings on actual behaviours. Finally, I selected and developed the dependent variables as those discussed in the literature to be affected by the wording. However, different, more systematic approaches are possible to pursue, such as protection motivation theory.^21^

To conclude, the words we use to communicate information about AMR matter.^12,22^ The use of the term ‘silent’ pandemic is consequential but only when its intended meaning is explicitly stated. Taken together, it is advisable to avoid using the term.

## Supplementary Material

dlae018_Supplementary_Data

## Data Availability

The datasets, codebook, R code and materials are available at: https://osf.io/8h3d5. Pre-registrations are available at: https://aspredicted.org/de2y9.pdf and https://aspredicted.org/n66fk.pdf.
