# Supplementary material for: Should we stop referring to the pandemic of antimicrobial resistance as silent?
Source: JAC Antimicrob Resist. 2024 Feb 7;6(1):dlae018. doi: 10.1093/jacamr/dlae018 (PMC10848890; doi:10.1093/jacamr/dlae018)
Supplement: dlae018_Supplementary_Data [file dlae018_supplementary_data.docx]

**Experiment 1: Materials**

**Pandemic Condition:**

*Please indicate your level of agreement or disagreement with the following statements concerning* ***the pandemic of antimicrobial resistance.***

The pandemic of antimicrobial resistance is an exceptionally serious health threat.

I am willing to raise more awareness about the pandemic of antimicrobial resistance.

The used scale:

Strongly disagree

Disagree

Somewhat disagree

Neither agree nor disagree

Somewhat agree

Agree

Strongly agree

**Silent Pandemic Condition:**

*Please indicate your level of agreement or disagreement with the following statements concerning* ***the*** ***silent pandemic of antimicrobial resistance.***

The silent pandemic of antimicrobial resistance is an exceptionally serious health threat.
I am willing to raise more awareness about the silent pandemic of antimicrobial resistance.

The used scale:

Strongly disagree

Disagree

Somewhat disagree

Neither agree nor disagree

Somewhat agree

Agree

Strongly agree

**Socio-demographic questions**

Almost done. Please answer the following questions.

What is your age?

What is your gender?

Male

Female

Other

Which of the following best describes your highest achieved education level?

Less than High School

Finished High School

Undergraduate Degree

Master's Degree

Doctoral (PhD) or Professional (JD, MD) Degree

What is your occupation?

Management, professional, and related

Service

Sales and office

Farming, fishing, and forestry

Construction, extraction, and maintenance

Production, transportation, and material moving

Government

Retired

Unemployed

Students

Other

What ethnic group do you belong to?

White

Black

Asian

Mixed

Other

**Reading Check Question**

*This task is simple, you must select the number eleven from the options below.*

Based solely on the text you read above, what is the number you should select?

7

13

11

1

100

**Experiment 2: Materials**

**Instructions**

Please carefully read the informative text provided on the next page. It contains important information relevant to the questions that will follow. Take your time to thoroughly understand the content before proceeding to answer the questions.

**Pandemic Condition:**

**The pandemic of antimicrobial resistance.**
 
Antimicrobial resistance occurs when bacteria, viruses, fungi and parasites change over time and no longer respond to medicines, making infections harder to treat and increasing the risk of disease spread, severe illness and death.
 
As a result of drug resistance, antibiotics and other antimicrobial medicines become ineffective and infections become increasingly difficult or impossible to treat.
 
The **pandemic** **of antimicrobial resistance** causes an estimated 1.3 million deaths every year. Without effective antimicrobials, the success of modern medicine in treating infections, including during major surgery and cancer chemotherapy, would be at increased risk.
 
 
*Please indicate your level of agreement or disagreement with the following statements.*Antimicrobial resistance is an exceptionally serious health threat.

I believe that the government should invest more money in addressing the issue of antimicrobial resistance.

I support strict policies that promote the judicious use of antimicrobials.

I am willing to actively raise more awareness about antimicrobial resistance.

I intend to use antimicrobials only as prescribed by a healthcare professional.

The used scale:

Strongly disagree

Disagree

Somewhat disagree

Neither agree nor disagree

Somewhat agree

Agree

Strongly agree

**Silent Pandemic Condition:**

**The silent pandemic of antimicrobial resistance.**
 
Antimicrobial resistance occurs when bacteria, viruses, fungi and parasites change over time and no longer respond to medicines, making infections harder to treat and increasing the risk of disease spread, severe illness and death.
 
As a result of drug resistance, antibiotics and other antimicrobial medicines become ineffective and infections become increasingly difficult or impossible to treat.
 
The **silent pandemic** **of antimicrobial resistance** causes an estimated 1.3 million deaths every year. Without effective antimicrobials, the success of modern medicine in treating infections, including during major surgery and cancer chemotherapy, would be at increased risk.
 
 
*Please indicate your level of agreement or disagreement with the following statements.*Antimicrobial resistance is an exceptionally serious health threat.

I believe that the government should invest more money in addressing the issue of antimicrobial resistance.

I support strict policies that promote the judicious use of antimicrobials.

I am willing to actively raise more awareness about antimicrobial resistance.

I intend to use antimicrobials only as prescribed by a healthcare professional.

The used scale:

Strongly disagree

Disagree

Somewhat disagree

Neither agree nor disagree

Somewhat agree

Agree

Strongly agree

**Socio-demographic questions**

The same questions as in Experiment 1 were used.

**Reading Check Question**The same question as in Experiment 1 was used.

**Experiment 3: Materials**

**Instructions**

The same questions as in Experiment 2 were used.

**Pandemic Condition:**

**The pandemic of antimicrobial resistance.**
 
Antimicrobial resistance occurs when bacteria, viruses, fungi and parasites change over time and no longer respond to medicines, making infections harder to treat and increasing the risk of disease spread, severe illness and death.
 
As a result of drug resistance, antibiotics and other antimicrobial medicines become ineffective and infections become increasingly difficult or impossible to treat.
 
The **pandemic** **of antimicrobial resistance** causes an estimated 1.3 million deaths every year. Without effective antimicrobials, the success of modern medicine in treating infections, including during major surgery and cancer chemotherapy, would be at increased risk.

**Silent Pandemic Condition:**

**The silent pandemic of antimicrobial resistance.**
 
**Antimicrobial resistance is termed a 'silent pandemic' because it spreads quietly and gradually around the world, receiving less attention than it should compared with other pandemics, despite being equally harmful to people's health.**
 
Antimicrobial resistance occurs when bacteria, viruses, fungi and parasites change over time and no longer respond to medicines, making infections harder to treat and increasing the risk of disease spread, severe illness and death.
 
As a result of drug resistance, antibiotics and other antimicrobial medicines become ineffective and infections become increasingly difficult or impossible to treat.
 
The **silent pandemic** **of antimicrobial resistance** causes an estimated 1.3 million deaths every year. Without effective antimicrobials, the success of modern medicine in treating infections, including during major surgery and cancer chemotherapy, would be at increased risk.

**The dependent measures:**

The same questions as in Experiment 2 were used.

**The reading recall questions:**

Which of the following statements was mentioned in the text you just read?

- Antimicrobial resistance is spreading loudly and rapidly, attracting a lot of attention.
- Antimicrobial resistance is termed a 'silent pandemic' because it quietly and gradually spreads around the world receiving less attention than it should.
- Antimicrobial resistance makes antibiotics and other medicines much more effective.
- The text did not mention anything regarding the attention given to antimicrobial resistance.

**Socio-demographic questions**

The same questions as in Experiment 1 were used.

**Reading check question**The same question as in Experiment 1 was used.
